# Development of overt hepatic encephalopathy increases mortality in patients with cirrhosis: a multicenter retrospective cohort study

**DOI:** 10.1007/s00535-025-02309-w

**Published:** 2025-10-17

**Authors:** Taisei Iwasa, Takao Miwa, Yuki Utakata, Mikita Oi, Mayu Asakura, Takumi Onishi, Masashi Aiba, Shinji Unome, Tatsunori Hanai, Makoto Shiraki, Seiji Adachi, Naoki Katsumura, Yasuhiro Kawashima, Shinji Nishiwaki, Masahito Shimizu

**Affiliations:** 1 Department of Internal Medicine, Seino Kosei Hospital, 293-1 Shimoiso, Ono-cho, Ibi-gun, Gifu, 501-0532 Japan; 2 Department of Gastroenterology/Internal Medicine, Graduate School of Medicine, Gifu University, 1-1 Yanagido, Gifu, 501-1194 Japan; 3 Department of Internal Medicine, Chuno Kosei Hospital, 5-1 Wakakusadori, Seki, Gifu, 501-3802 Japan; 4 Department of Gastroenterology, Nagoya Central Hospital, 3-7-7 Taiko, Nakamura-ku, Nagoya, Aichi 453-0801 Japan

**Keywords:** Albumin, Ammonia, Covert hepatic encephalopathy, Minimal hepatic encephalopathy, Sarcopenia, Survival

## Abstract

**Background:**

Overt hepatic encephalopathy (OHE) is a severe complication of liver cirrhosis. However, data on its incidence, prognostic significance, and associated risk factors in patients without OHE at baseline remain limited.

**Methods:**

A multicenter retrospective cohort study was conducted by reviewing records of hospitalized patients with cirrhosis at three institutions in Japan. OHE was defined as West Haven grade ≥ 2 and its incidence during the follow-up was estimated using the cumulative incidence function. Prognostic factors were assessed using Cox proportional hazards regression analysis, with OHE and hepatocellular carcinoma (HCC) development treated as time-dependent covariates. Independent predictors for OHE development were analyzed using fine-gray proportional hazards regression analysis.

**Results:**

Among 652 patients, the median age was 67 years, and 53% were male. The median model for end-stage liver disease (MELD) score was 9. During a median follow-up period of 3.2 years, 136 patients (21%) developed OHE and 183 patients (28%) died. The cumulative incidence of OHE at 1, 3, and 5 years was 8%, 16%, and 20%, respectively. Multivariable analysis demonstrated that OHE development (hazard ratio [HR], 3.07; 95% confidence interval [CI], 1.99–4.75) was a significant independent prognostic factor, regardless of age, sex, liver functional reserve, and HCC development. Furthermore, multivariable analysis identified lower body mass index, higher MELD score, lower albumin levels, and higher ammonia levels as independent predictors for OHE development.

**Conclusions:**

OHE development is common and increases mortality among patients with cirrhosis. Therefore, close monitoring of high-risk populations is warranted for early management of OHE.

**Supplementary Information:**

The online version contains supplementary material available at 10.1007/s00535-025-02309-w.

## Introduction

Liver cirrhosis, the end stage of chronic liver disease, is caused by factors such as hepatitis virus infection, obesity, type 2 diabetes, and alcohol abuse, among others, and represents a significant global health issue [1]. In 2019, cirrhosis was responsible for 2.4% of global deaths, with the number of associated deaths reaching 1,472,000—an increase of 10% compared to 2010 [1, 2]. The prognosis for patients with cirrhosis significantly deteriorates as the disease progresses from compensated to decompensated stages, leading to various complications. Identifying and preventing these complications in high-risk individuals is crucial to improving outcomes for these patients.

Hepatic encephalopathy (HE) is a common and severe complication of cirrhosis that significantly impacts the clinical outcomes of patients [3, 4]. According to Western studies, 10–21% of patients with cirrhosis present with overt HE (OHE) at the time of diagnosis, 30–40% will experience at least one episode of OHE during their clinical course, and 40% of those with a previous episode will experience a recurrence [5]. In a study focusing on hospitalized patients with decompensated cirrhosis, OHE was responsible for nearly half of the readmissions [5]. These reports offer valuable epidemiological data on OHE and its clinical impact on outcomes in patients with cirrhosis. However, most of these studies included patients who already had OHE at baseline, with very few examining its "development" and impact on mortality among patients without prior OHE. Additionally, epidemiological data on OHE are predominantly based on Western studies, leaving a gap in knowledge regarding Japanese patients with cirrhosis. Furthermore, identifying independent predictors for the development of OHE is essential for the early detection of high-risk populations and the implementation of preventive and therapeutic measures to improve mortality rates in this group.

In view of the above clinical issues, we conducted a new retrospective cohort study on HE in Japanese patients with cirrhosis. The primary aim of this study was to investigate the incidence of OHE development and its impact on mortality among Japanese patients with cirrhosis. The secondary aim was to identify the risk factors for OHE development in this population.

## Methods

### Study protocol

This retrospective cohort study included Japanese patients with cirrhosis who were admitted to one of three institutions in Japan—Gifu University Hospital, Chuno Kosei Hospital, or Nagoya Central Hospital—between March 2004 and December 2023. The study protocol was reviewed and approved by the Institutional Review Board of Gifu University Graduate School of Medicine (approval number: 2024–226). This study was conducted in accordance with the principles of the 2013 Declaration of Helsinki. Due to the retrospective nature of the study, informed consent was obtained using an opt-out method.

### Participants and follow-up

The inclusion criteria for this study were patients with cirrhosis of any etiology, aged 18 years or older, admitted between March 2004 and December 2023. Cirrhosis was diagnosed based on clinical features, including complications, liver histology, laboratory data, and medical imaging. Exclusion criteria included organ transplantation such as liver, active malignancies including hepatocellular carcinoma (HCC), history of OHE, large portosystemic shunts, portal vein thrombosis, estimated glomerular filtration rate < 15 mL/min/1.73 m^2^, dialysis, life-threatening comorbidities, and an opt-out refusal. These criteria were applied to minimize bias by considering factors that could influence the development of OHE or mortality in patients with cirrhosis. After discharge, patients were followed up in the outpatient clinic at least every three months and treated according to the Japanese guidelines for cirrhosis [6, 7].

### Diagnosis and grading of HE

HE was diagnosed and graded according to the West Haven criteria, with the development of OHE defined as the occurrence of HE ≥ grade 2 during the clinical course [3]. Patients were followed until the last visit, death, or August 2, 2024, whichever came first.

### Data collection

The following baseline data were collected from medical records: age, sex, weight, height, etiology of cirrhosis, comorbidities including diabetes mellitus, hypertension, heart failure, respiratory failure, ascites, hepatic encephalopathy, and laboratory data. Body mass index (BMI), Child–Pugh score, model for end-stage liver disease (MELD) score, and estimated glomerular filtration rate were automatically calculated from the obtained data. The cutoff value for each variable was based on the normative value in previous reports [6–9]. The demographic variables were assessed at the time of admission, and biochemical parameters were measured on the day of admission or the following day under fasting conditions. Biochemical analyses were performed under fasting condition to ensure the reproducibility of serum ammonia measurement [10]. For outcomes, the dates of OHE, HCC, and death were recorded. The time from enrolment to each event and the time from OHE development to death were calculated from each date.

### Statistical analysis

Data were presented as medians with interquartile ranges for continuous variables and as numbers with percentages for categorical variables. Baseline characteristics of the groups were compared using the Mann–Whitney *U* test or chi-square test. Survival curves were estimated using the Kaplan–Meier method, and differences between groups were assessed using the log-rank test. Independent factors for survival were evaluated using the Cox proportional hazards regression model, with results expressed as hazard ratios (HRs) with 95% confidence intervals (CIs). Multiple pairwise comparisons were conducted using the Bonferroni correction. Multivariable analysis incorporated OHE and HCC development as time-dependent covariates to explore the association between event occurrence and mortality [11]. Considering mortality as a competing risk, cumulative incidence curves for OHE were estimated using the cumulative incidence function, and groups were compared using Gray’s test. Independent factors for OHE development were evaluated using the Fine–Gray competing risk regression model, with results presented as subdistribution hazard ratios (SHRs) with 95% CIs. Covariates were predetermined based on the clinical importance and multicollinearity of each variable. Patients with missing values were excluded from the analyses; therefore, no data imputation was performed. A two-sided *p* < 0.05 was set as the threshold for statistical significance. Analyses were performed using JMP Pro (version 17.0.0; SAS Institute Inc., Cary, NC, USA) and R (version 4.3.1; R Foundation for Statistical Computing, Vienna, Austria).

## Results

### Baseline characteristics of patients with cirrhosis

Of the 908 patients reviewed (796 at Gifu University, 83 at Chuno Kosei Hospital, and 29 at Nagoya Central Hospital), 652 were included in the analysis (Supplementary Fig. 1). The baseline characteristics of the included patients are summarized in Table 1. The median age was 67 years, 53% were male, and the median BMI was 23.3 kg/m^2^. Ascites was present in 41% of patients and the median Child–Pugh and MELD scores were 7 and 9, respectively. The most common etiology of cirrhosis was viral hepatitis (42%), followed by alcohol-associated/related liver disease (27%), metabolic dysfunction-associated steatotic liver disease (8%), and other causes (23%).

**Table 1:** Baseline characteristics of patients with liver cirrhosis divided by OHE development

Characteristic	All patients	OHE	No OHE	*p-*value*
	(*n* = 652)	(*n* = 136)	(*n* = 516)	
Age (years)	67 (57–74)	66 (56–72)	67 (57–74)	0.332
Male, n (%)	343 (53)	73 (54)	270 (52)	0.854
Body mass index (kg/m^2^)	23.3 (21.2–25.7)	23.1 (21.4–25.4)	23.4 (21.1–25.7)	0.785
Etiology (Viral/ALD/MASLD/Others), n	276/173/50/153	61/36/9/30	215/137/41/123	0.885
Diabetes mellitus, n (%)	172 (26)	36 (27)	136 (26)	1.000
Ascites, n (%)	270 (41)	82 (60)	188 (36)	< 0.001
Varices, n (%)	406 (62)	110 (81)	296 (57)	< 0.001
Child–Pugh class (A/B/C)	384/182/86	47/56/33	337/126/53	< 0.001
Child–Pugh score	7 (6–9)	8 (7–10)	6 (5–9)	< 0.001
MELD score	9 (7–11)	11 (9–14)	8 (7–11)	< 0.001
International normalized ratio	1.11 (1.02–1.26)	1.21 (1.10–1.37)	1.08 (1.01–1.22)	< 0.001
Platelet (10^9^/L)	95 (55–137)	72 (45–103)	102 (61–143)	< 0.001
Creatinine (mg/dL)	0.71 (0.59–0.89)	0.75 (0.63–0.98)	0.71 (0.59–0.87)	0.076
Albumin (g/dL)	3.3 (2.7–3.9)	2.9 (2.4–3.3)	3.5 (2.7–3.9)	< 0.001
Bilirubin (mg/dL)	1.1 (0.8–1.7)	1.4 (1.0–2.4)	1.1 (0.8–1.6)	< 0.001
Sodium (meq/L)	139 (137–141)	139 (137–141)	139 (137–140)	0.193
Ammonia (mcg/dL)	58 (41–84)	76 (52–102)	55 (39–78)	< 0.001
BCAA, n (%)	268 (41)	81 (60)	187 (36)	< 0.001
Lactulose, n (%)	62 (10)	21 (15)	41 (8)	0.013
Rifaximin, n (%)	25 (4)	11 (8)	14 (3)	0.009

Values are presented as numbers (percentage) or medians (interquartile range)

^*^Groups were compared using the chi-square test or Mann–Whitney *U* test

*ALD* alcohol-related liver disease, *BCAA* branched chain amino acid, *MELD* model for end-stage liver disease, *MASLD* metabolic dysfunction-associated steatotic liver disease, *OHE* overt hepatic encephalopathy

The comparison between patients with and without OHE development is also presented in Table 1. Patients who developed OHE had significantly worse liver functional reserves, as evidenced by a greater presence of ascites, higher Child–Pugh and MELD scores, higher international normalized ratios, lower platelet counts, lower levels of serum albumin, and higher levels of bilirubin and ammonia compared to those without OHE.

### Incidence of OHE in patients with cirrhosis

During a median follow-up period of 3.2 years (interquartile range, 1.2–5.6), 21% (*n* = 136) of patients developed OHE and 16% (*n* = 102) developed HCC. Among those who developed OHE, 68% (*n* = 93) had grade 2, 25% (*n* = 34) had grade 3, and 7% (*n* = 9) had grade 4. The cumulative incidence rates of OHE at 1, 3, and 5 years were 8%, 16%, and 20%, respectively. The cumulative incidence of OHE at 1, 3, and 5 years was 8%, 14%, and 17% in viral hepatitis, 8%, 16%, and 21% in alcohol-associated/related liver disease, and 9%, 20%, and 23% in metabolic dysfunction-associated steatotic liver disease, respectively.

### Impact of OHE development on mortality in patients with cirrhosis

**Fig. 1:**
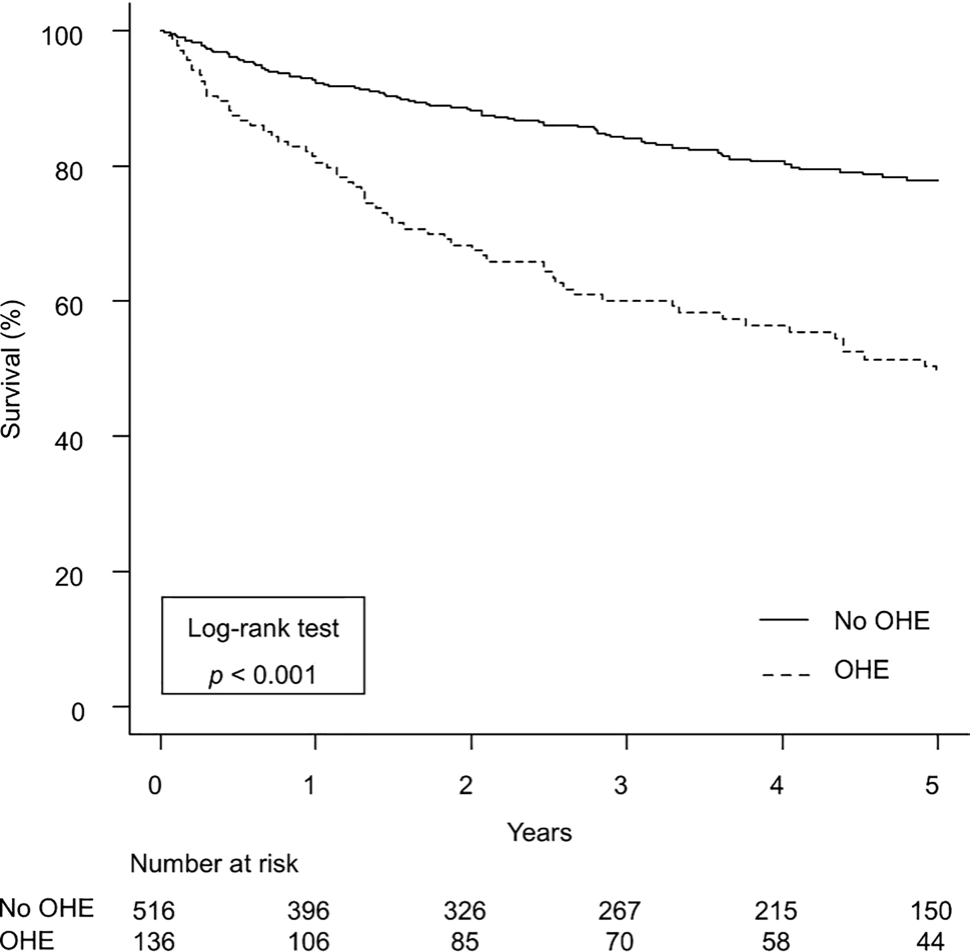
Overall survival of patients with cirrhosis according to OHE development. *OHE* overt hepatic encephalopathy

During the follow-up period, 28% (*n* = 183) of patients died of liver failure (*n* = 115; 63%), HCC (*n* = 28; 15%), and other causes (*n* = 40; 22%). The overall survival rates at 1, 3, and 5 years were 90%, 78%, and 71%, respectively. The adjusted HRs for mortality in patients with cirrhosis are shown in Table 2. Considering OHE and HCC development as time-dependent covariates**,** OHE development (HR 3.07; 95% CI 1.99–4.75; *p* < 0.001) was a significant determinant of mortality, independent of age, sex, etiology of cirrhosis, liver functional reserves, and HCC development. Similar results were obtained in a model including medications (Supplementary Table 1). The overall survival rates of patients with OHE development were significantly lower than those without (at 1, 3, and 5 years: 81%, 60%, and 49% vs. 93%, 84%, and 78%, respectively; *p* < 0.001; Fig. 1). In addition, survival after the first episode of OHE was also evaluated according to the second OHE episode (Supplementary Fig. 2).

**Table 2:** Prognosis prediction model including time-dependent covariates in patients with cirrhosis

Characteristic	HR (95% CI)	*p* value^*^
*Baseline covariates*		
Age	1.04 (1.02–1.05)	< 0.001
Male	2.12 (1.47–3.07)	< 0.001
Body mass index (kg/m^2^)	1.04 (1.00–1.08)	0.050
Etiology of cirrhosis		
Viral^a^	1.00	
ALD	1.67 (1.13–2.46)	0.010
MASLD	0.86 (0.33–2.22)	0.751
Others	2.20 (1.41–3.42)	< 0.001
Ascites	1.83 (1.22–2.74)	0.003
Varices	1.02 (0.69–1.51)	0.919
MELD score	1.05 (1.02–1.08)	0.004
Platelet (10^9^/L)	1.00 (0.99–1.00)	0.187
Albumin (g/dL)	0.64 (0.48–0.84)	0.001
Ammonia (mcg/dL)	1.00 (0.99–1.00)	0.936
*Time dependent covariates*		
OHE development	3.07 (1.99–4.75)	< 0.001
HCC development	4.46 (2.86–6.97)	< 0.001

^*^Multivariable analysis was performed using the Cox proportional hazard model

^a^Reference group

*ALD* alcohol-associated/related liver disease; *CI* confidence interval; *HCC* hepatocellular carcinoma, *HR* hazard ratio, *MASLD* metabolic dysfunction-associated steatotic liver disease, *MELD* model for end-stage liver disease, OHE overt hepatic encephalopathy

### Impact of OHE grade on mortality in patients with cirrhosis

**Fig. 2:**
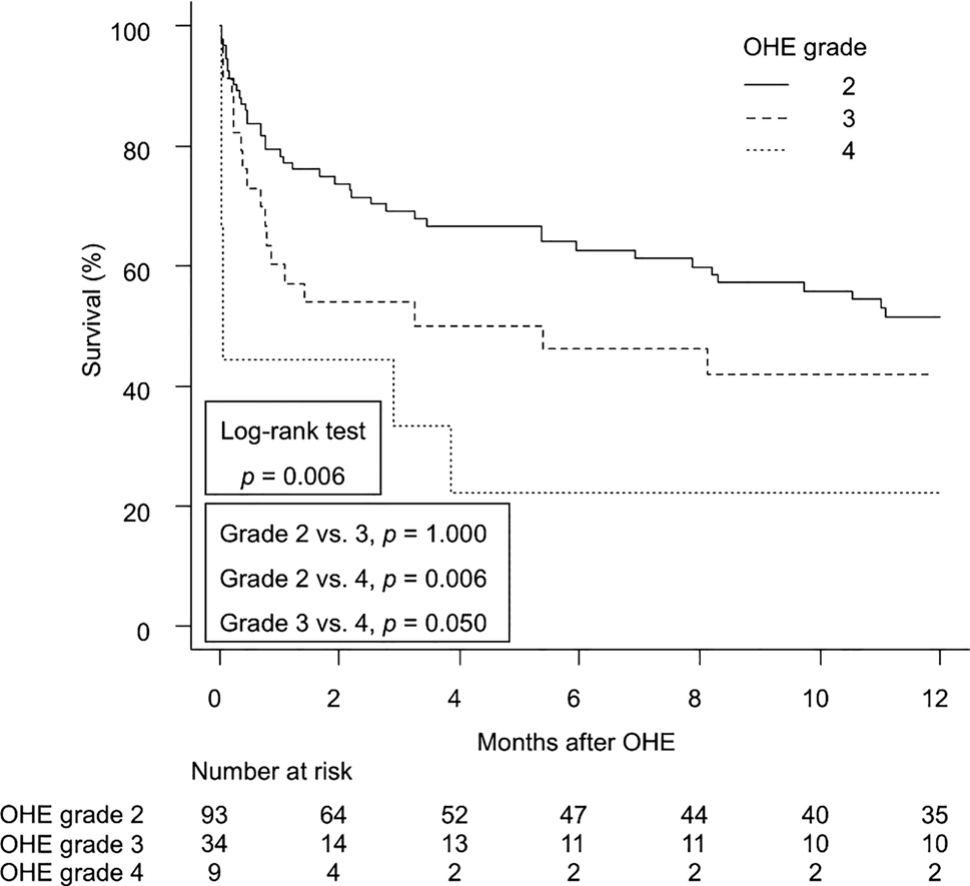
Survival of patients with cirrhosis after OHE development divided by OHE grade. *OHE* overt hepatic encephalopathy

A subgroup analysis of patients who developed OHE (*n* = 136) was conducted to investigate the impact of OHE grade on mortality in patients with cirrhosis. Regarding mortality, a significant difference was observed between patients with OHE grade 4 and grade 2 (HR 2.99; 95% CI 1.47–6.07; *p* = 0.003), and between grade 4 and grade 3 (HR 2.65; 95% CI 1.20–5.83; *p* = 0.016). However, no significant difference was found between grade 3 and grade 2 (HR 1.13; 95% CI 0.68–1.86; *p* = 0.636). The survival curve also indicated a significant effect of OHE grade on mortality following OHE development in patients with cirrhosis, with 1-year survival rates after OHE development being 22% for grade 4, 42% for grade 3, and 52% for grade 2 (*p* = 0.006; Fig. 2).

### Independent factors of OHE development in patients with cirrhosis

**Fig. 3:**
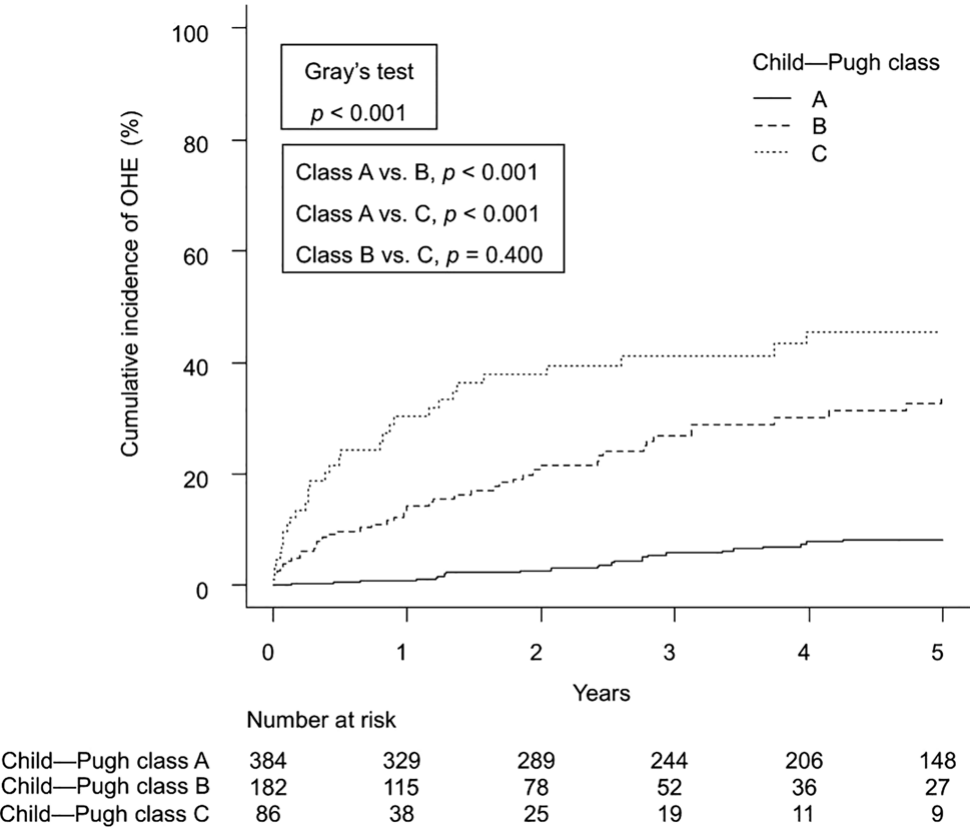
Cumulative incidence of OHE in patients with cirrhosis divided by Child–Pugh class. *OHE* overt hepatic encephalopathy

The adjusted SHRs for determinants of OHE development are presented in Table 3. In patients with cirrhosis, BMI (SHR 0.93; 95% CI 0.89–0.98; *p* = 0.004), varices (SHR 2.14; 95% CI 1.31–3.48; *p* = 0.002), MELD score (SHR 1.06; 95% CI 1.03–1.10; *p* < 0.001), serum albumin (SHR 0.61; 95% CI 0.44–0.84; *p* = 0.003), and ammonia levels (SHR 1.01; 95% CI 1.00–1.01; *p* < 0.001) were identified as independent factors for OHE development. Similar results were obtained in a model including medications (Supplementary Table 2). Factors associated with OHE development in each etiology are shown in Supplementary Table 3. The cumulative incidence of OHE was significantly stratified by high MELD scores (15 points; *p* < 0.001), low albumin levels (3.5 g/dL; *p* < 0.001), and high ammonia levels (80 mg/dL; *p* < 0.001, Supplementary Fig. 3). Regarding the Child–Pugh class, the cumulative incidences of OHE at 1, 3, and 5 years were 1%, 6%, and 8% for class A; 14%, 27%, and 34% for class B; and 30%, 41%, and 46% for class C (*p* < 0.001; Fig. 3).

**Table 3:** OHE prediction model in patients with cirrhosis

Characteristic	SHR (95% CI)	*p-*value*
Age (years)	0.99 (0.98–1.01)	0.430
Male	0.83 (0.56–1.23)	0.350
Body mass index (kg/m^2^)	0.93 (0.89–0.98)	0.004
Etiology of cirrhosis		
Viral^a^	1.00	
ALD	0.71 (0.44–1.14)	0.160
MASLD	1.70 (0.79–3.69)	0.180
Others	1.56 (0.98–2.48)	0.059
Ascites	1.29 (0.81–2.06)	0.290
Varices	2.14 (1.31–3.48)	0.002
MELD score	1.06 (1.03–1.10)	< 0.001
Platelet (10^9^/L)	1.00 (0.99–1.00)	0.620
Albumin (g/dL)	0.61 (0.44–0.84)	0.003
Ammonia (mcg/dL)	1.01 (1.00–1.01)	< 0.001

^*^Multivariable analysis was performed using the Fine–Gray competing risk regression model

*ALD* alcohol-associated/related liver disease, *BCAA* branched chain amino acid, *CI* confidence interval, *MASLD* metabolic dysfunction-associated steatotic liver disease, *MELD* model for end-stage liver disease, *OHE* overt hepatic encephalopathy, *SHR* subdistribution hazard ratio

## Discussion

OHE is a devastating complication of cirrhosis. However, few longitudinal studies have examined the prognostic impact of its development in patients with cirrhosis. The results of this study clearly demonstrated that OHE development significantly worsened mortality among Japanese patients with cirrhosis. Additionally, this study provided clear epidemiological data on the incidence and factors associated with its development in these patients.

The first significant finding of this study was the prognostic importance of OHE development in patients with cirrhosis. A study from Taiwan showed that the development of complications related to cirrhosis, including OHE, is significantly associated with increased mortality [12]. Similarly, in the United States, the development of OHE is linked to higher mortality rates in patients with compensated cirrhosis (Child–Pugh class A/B) [11]. Therefore, OHE is considered a major risk factor for mortality in patients with cirrhosis [12, 13]. However, no studies have specifically focused on the prognostic impact of OHE “development” in patients with cirrhosis, particularly within the Japanese cohort. This is the first report to demonstrate that the new development of OHE is a prognostic factor in these patients. The inclusion of time-dependent covariates is a well-established method for investigating the association between event occurrence and outcomes [11]. The results of this analysis using this method revealed that in a Japanese cohort of cirrhosis patients without an episode of OHE at baseline, those who developed OHE during the clinical course were three times more likely to die than those who did not. Additionally, patients who developed grade 4 OHE had a significantly higher mortality rate than those who developed grade 3 or 2 OHE. These results are consistent with a previous study indicating that patients awaiting liver transplantation with grade 3/4 or grade 1/2 OHE have lower survival rates than those without OHE [13]. The development and severity of OHE are critical determinants of prognosis in patients with cirrhosis.

The second important finding of this study was the epidemiological data on the incidence and risk factors for OHE development in patients with cirrhosis. In a previous study, the 1-year probability of developing OHE in U.S. patients with cirrhosis was 14%, with patients in Child–Pugh class B experiencing a significantly higher incidence (25%) compared to those in class A (10%) [14]. A plausible explanation for the lower incidence of OHE in the present study relative to the previous report could be the relatively preserved liver functional reserves and the early detection and intervention for covert HE before it progresses to OHE in our cohort [15]. Regarding the risk factors for OHE development, lower BMI, higher MELD score, hypoalbuminemia, and hyperammonemia were identified as independent factors in patients with cirrhosis. Low BMI is indicative of sarcopenia, which is an established risk factor for OHE in patients with cirrhosis [15]. Additionally, higher MELD scores, hypoalbuminemia, and hyperammonemia are well-known risk factors for OHE development in these patients [16–19]. This robust evidence from a multicenter Japanese cohort study suggests that patients with these risk factors should be closely monitored with the goal of early detection and prevention of OHE to improve survival outcomes.

Furthermore, given that advanced stages of HE are associated with worse clinical outcomes, as demonstrated in this study, early intervention for HE should be considered in daily practice. Indeed, managing HE with treatments such as lactulose or rifaximin, which can reduce serum ammonia levels, is known to improve survival in patients with cirrhosis [18, 19]. Nutritional therapy, the fundamental treatment for sarcopenia and hypoalbuminemia, also serves as a treatment for HE. Specifically, nutritional therapy that includes branched-chain amino acid supplementation has been shown to improve HE and the prognosis of patients with cirrhosis [20]. Therefore, clinicians should identify the high-risk population for developing OHE and employ these preventive measures before the condition becomes fatal.

This study has several limitations. First, focusing on a Japanese cohort may limit the generalizability of the results to other regions. Second, the retrospective nature of the study cannot exclude the possibility of bias. However, we do not believe this to be a major concern, as we utilized a multicenter cohort with prospectively collected data, and the method of follow-up was not influenced by the retrospective design. Third, other liver decompensation events and medications can influence the outcomes of patients with cirrhosis. Fourth, patients with the second episode of OHE had significantly better survival than those without due to the immortal time bias [21]. Therefore, the impact of OHE recurrence on mortality should be evaluated in future studies including many patients with OHE at baseline. Additionally, it should be emphasized that the study has notable strengths, including its multicenter approach and an adequate sample size and number of events to ensure robust analysis.

In conclusion, this study provides strong evidence that the development of OHE adversely affects the prognosis of Japanese patients with cirrhosis. Additionally, it offers valuable epidemiological data on the incidence and predictors of OHE development in these patients. Given that OHE development is associated with higher mortality, high-risk patients should be monitored closely, and early detection and intervention for OHE should be prioritized in the management of cirrhosis.

## Supplementary Information

Below is the link to the electronic supplementary material.

Supplementary file1 (DOCX 72 KB)

Supplementary file2 (DOCX 43 KB)

Supplementary file3 (DOCX 19 KB)

Supplementary file4 (DOCX 18 KB)

Supplementary file5 (DOCX 20 KB)
