# Supplementary material for: Development of overt hepatic encephalopathy increases mortality in patients with cirrhosis: a multicenter retrospective cohort study
Source: J Gastroenterol. 2025 Oct 17;61(1):78–84. doi: 10.1007/s00535-025-02309-w (PMC12791057; doi:10.1007/s00535-025-02309-w)

**Figure S1.** Flow diagram of eligibility criteria. Abbreviations: eGFR, estimated glomerular filtration rate; OHE, overt hepatic encephalopathy


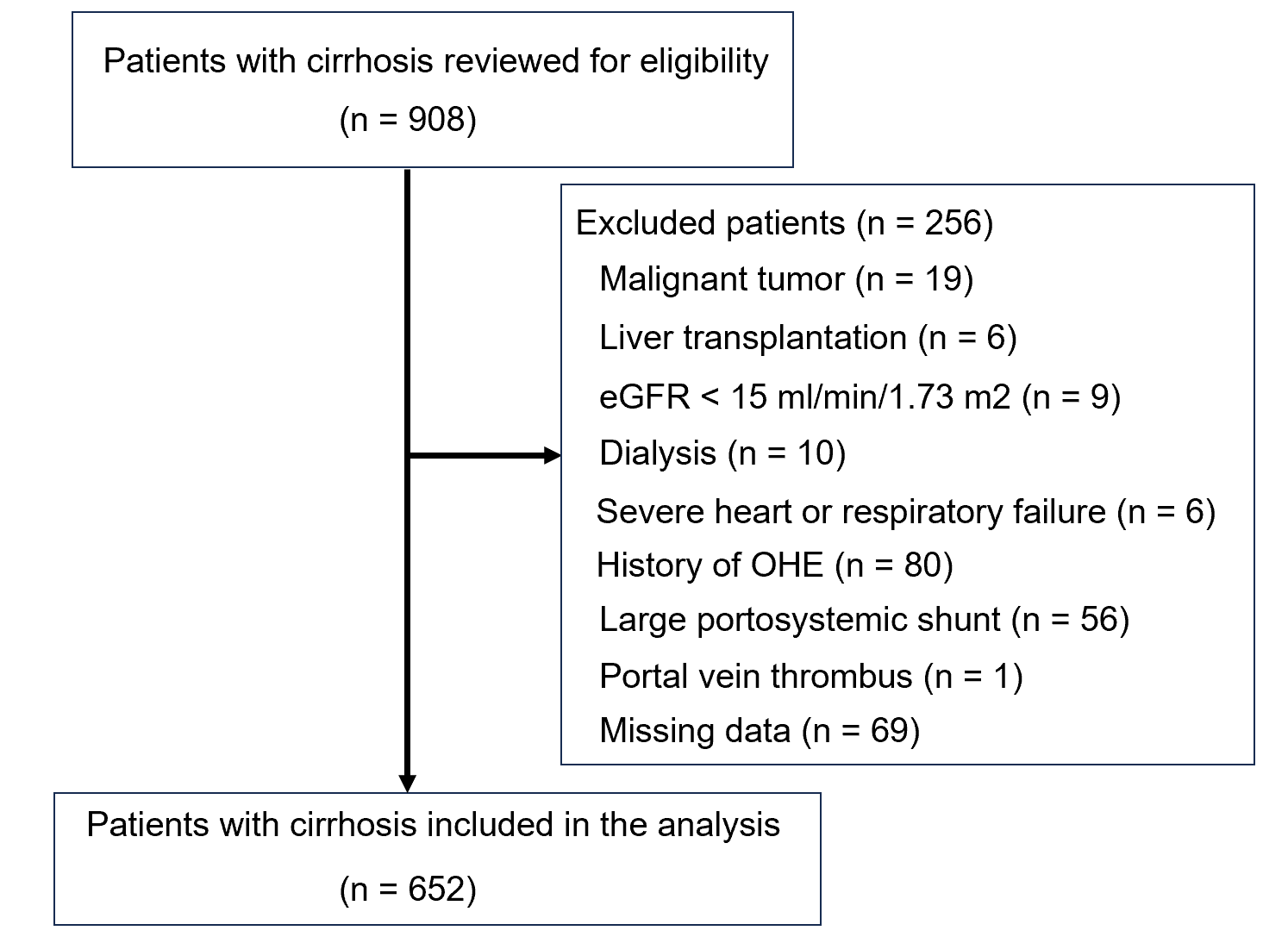

Supplement: Supplementary file 1 — Supplementary file1 (DOCX 72 KB) [file 535_2025_2309_MOESM1_ESM.docx]
