# Supplementary material for: Development of overt hepatic encephalopathy increases mortality in patients with cirrhosis: a multicenter retrospective cohort study
Source: J Gastroenterol. 2025 Oct 17;61(1):78–84. doi: 10.1007/s00535-025-02309-w (PMC12791057; doi:10.1007/s00535-025-02309-w)

**Figure S2.** Survival of patients with cirrhosis after OHE development divided by OHE recurrence. Abbreviation: OHE, overt hepatic encephalopathy


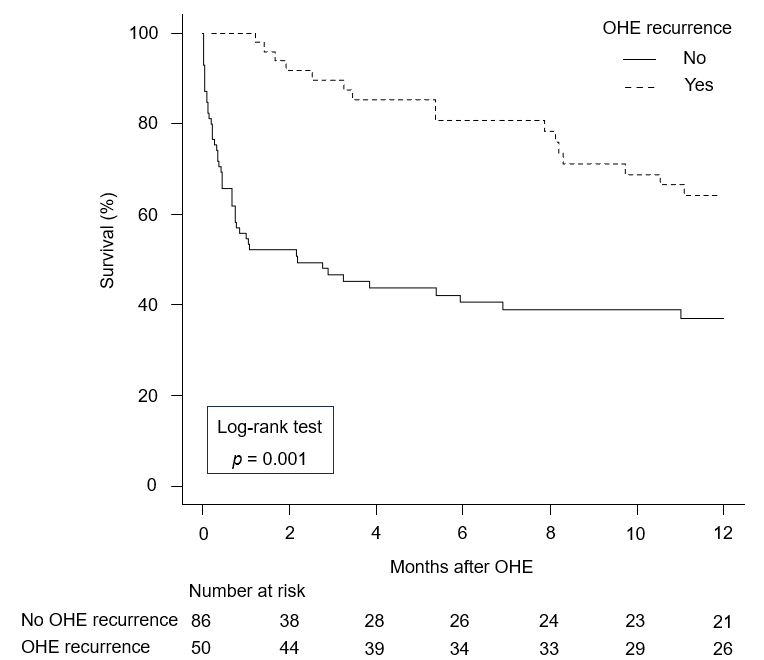

Supplement: Supplementary file 2 — Supplementary file2 (DOCX 43 KB) [file 535_2025_2309_MOESM2_ESM.docx]
