# Supplementary material for: Development of overt hepatic encephalopathy increases mortality in patients with cirrhosis: a multicenter retrospective cohort study
Source: J Gastroenterol. 2025 Oct 17;61(1):78–84. doi: 10.1007/s00535-025-02309-w (PMC12791057; doi:10.1007/s00535-025-02309-w)
Supplement: Supplementary file 3 — Supplementary file3 (DOCX 19 KB) [file 535_2025_2309_MOESM3_ESM.docx]

Supplementary Table 1. Prognosis prediction model including medications in patients with cirrhosis

| Characteristic | HR (95% CI) | *p*-value^*^ |
| --- | --- | --- |
| Baseline covariates |  |  |
| Age | 1.04 (1.02–1.05) | <0.001 |
| Male | 2.17 (1.50–3.13) | <0.001 |
| Body mass index (kg/m^2^) | 1.04 (0.99–1.08) | 0.078 |
| Etiology of cirrhosis |  |  |
| Viral^a^ | 1.00 |  |
| ALD | 1.59 (1.08–2.35) | 0.020 |
| MASLD | 0.89 (0.34–2.32) | 0.812 |
| Others | 2.12 (1.37–3.28) | <0.001 |
| Ascites | 1.86 (1.22–2.84) | 0.004 |
| Varices | 1.04 (0.70–1.54) | 0.860 |
| MELD score | 1.04 (1.01–1.08) | 0.010 |
| Platelet (10^9^/L) | 1.00 (0.99–1.00) | 0.209 |
| Albumin (g/dL) | 0.59 (0.43–0.80) | <0.001 |
| Ammonia (mcg/dL) | 1.00 (0.99–1.00) | 0.950 |
| Medications |  |  |
| BCAA | 0.89 (0.59–1.33) | 0.561 |
| Lactulose | 0.70 (0.44–1.12) | 0.134 |
| Rifaximin | 0.50 (0.18–1.40) | 0.186 |
| Time dependent covariates |  |  |
| OHE development | 3.24 (2.88–5.02) | <0.001 |
| HCC development | 4.48 (2.88–6.98) | <0.001 |

*Multivariable analysis was performed using the Cox proportional hazard model.

^a^Reference group

Abbreviations: ALD, alcohol-associated/related liver disease; BCAA, branched chain amino acid; CI, confidence interval; HCC, hepatocellular carcinoma; HR, hazard ratio; MASLD, metabolic dysfunction-associated steatotic liver disease; MELD, model for end-stage liver disease; OHE, overt hepatic encephalopathy
