# Supplementary material for: Development of overt hepatic encephalopathy increases mortality in patients with cirrhosis: a multicenter retrospective cohort study
Source: J Gastroenterol. 2025 Oct 17;61(1):78–84. doi: 10.1007/s00535-025-02309-w (PMC12791057; doi:10.1007/s00535-025-02309-w)
Supplement: Supplementary file 4 — Supplementary file4 (DOCX 18 KB) [file 535_2025_2309_MOESM4_ESM.docx]

Supplementary Table 2. OHE prediction model in patients with cirrhosis including medications

| Characteristic | SHR (95% CI) | *p-*value* |
| --- | --- | --- |
| Age (years) | 0.99 (0.98–1.01) | 0.500 |
| Male | 0.85 (0.57–1.27) | 0.420 |
| Body mass index (kg/m^2^) | 0.93 (0.88–0.97) | 0.002 |
| Etiology of cirrhosis |  |  |
| Viral^a^ | 1.00 |  |
| ALD | 0.68 (0.42–1.11) | 0.120 |
| MASLD | 1.45 (0.65–3.22) | 0.370 |
| Others | 1.53 (0.95–2.45) | 0.078 |
| Ascites | 1.23 (0.76–1.99) | 0.410 |
| Varices | 2.05 (1.23–3.39) | 0.006 |
| MELD score | 1.06 (1.02–1.10) | <0.001 |
| Platelet (10^9^/L) | 1.00 (0.99–1.00) | 0.650 |
| Albumin (g/dL) | 0.64 (0.46–0.90) | 0.009 |
| Ammonia (mcg/dL) | 1.01 (1.00–1.01) | <0.001 |
| Medications |  |  |
| BCAA | 1.11 (0.69–1.76) | 0.680 |
| Lactulose | 1.09 (0.61–1.98) | 0.770 |
| Rifaximin | 2.49 (1.16–5.31) | 0.018 |

*Multivariate analysis was performed using the Fine–Gray competing risk regression model.

Abbreviations: ALD, alcohol-associated/related liver disease; BCAA, branched chain amino acid; CI, confidence interval; MASLD, metabolic dysfunction-associated steatotic liver disease; MELD, model for end-stage liver disease; OHE, overt hepatic encephalopathy; SHR, subdistribution hazard ratio.
