# Supplementary material for: Development of overt hepatic encephalopathy increases mortality in patients with cirrhosis: a multicenter retrospective cohort study
Source: J Gastroenterol. 2025 Oct 17;61(1):78–84. doi: 10.1007/s00535-025-02309-w (PMC12791057; doi:10.1007/s00535-025-02309-w)
Supplement: Supplementary file 5 — Supplementary file5 (DOCX 20 KB) [file 535_2025_2309_MOESM5_ESM.docx]

Supplementary Table 3. Association between OHE development and each variables according to etiology of cirrhosis

| Characteristic | SHR (95% CI) | *p-*value* |
| --- | --- | --- |
| Viral (n = 276) |  |  |
| Age (years) | 1.01 (0.99–1.04) | 0.200 |
| Male | 1.07 (0.65–1.76) | 0.790 |
| Body mass index (kg/m^2^) | 0.99 (0.94–1.05) | 0.780 |
| Ascites | 3.92 (2.36–6.49) | <0.001 |
| MELD score | 1.04 (1.00–1.08) | 0.035 |
| Platelet (10^9^/L) | 0.99 (0.98–0.99) | <0.001 |
| Albumin (g/dL) | 0.37 (0.26–0.53) | <0.001 |
| Ammonia (mcg/dL) | 1.01 (1.00–1.01) | <0.001 |
| ALD (n = 173) |  |  |
| Age (years) | 0.97 (0.94–1.00) | 0.078 |
| Male | 1.07 (0.65–1.76) | 0.790 |
| Body mass index (kg/m^2^) | 0.94 (0.84–1.05) | 0.240 |
| Ascites | 2.25 (1.16–4.36) | 0.016 |
| MELD score | 1.07 (1.01–1.15) | 0.030 |
| Platelet (10^9^/L) | 1.00 (0.99–1.00) | 0.360 |
| Albumin (g/dL) | 0.66 (0.43–1.03) | 0.066 |
| Ammonia (mcg/dL) | 1.01 (0.99–1.01) | 0.210 |
| MASLD (n = 50) |  |  |
| Age (years) | 0.99 (0.95–1.04) | 0.760 |
| Male | 1.17 (0.24–5.69) | 0.840 |
| Body mass index (kg/m^2^) | 1.00 (0.93–1.09) | 0.920 |
| Ascites | 2.50 (0.64–9.72) | 0.190 |
| MELD score | 1.98 (1.46–2.68) | <0.001 |
| Platelet (10^9^/L) | 1.00 (0.99–1.02) | 0.750 |
| Albumin (g/dL) | 0.43 (0.25–9.77) | 0.004 |
| Ammonia (mcg/dL) | 1.93 (0.47–7.97) | 0.360 |

*Univariable analysis was performed using the Fine–Gray competing risk regression model.

Abbreviations: ALD, alcohol-related liver disease; BCAA, branched chain amino acid; CI, confidence interval; MASLD, metabolic dysfunction-associated steatotic liver disease; MELD, model for end-stage liver disease; OHE, overt hepatic encephalopathy; SHR, subdistribution hazard ratio.
